# Substantial and Sustained Improvements in Blood Pressure, Weight and Lipid Profiles from a Carbohydrate Restricted Diet: An Observational Study of Insulin Resistant Patients in Primary Care

**DOI:** 10.3390/ijerph16152680

**Published:** 2019-07-26

**Authors:** David J. Unwin, Simon D. Tobin, Scott W. Murray, Christine Delon, Adrian J. Brady

**Affiliations:** 1 General Practice, Norwood Surgery, Southport PR9 7EG, UK; 2 Department of Cardiology, Royal Liverpool University Hospital, Liverpool Centre for Cardiovascular Science, Liverpool L7 8XP, UK; 3 Independent Researcher, Data and Research Analyst, London, UK; 4 Department of Cardiology, University of Glasgow, Glasgow G4 0SF, UK

**Keywords:** hypertension, low carbohydrate diet, sodium, essential hypertension, lifestyle medicine, obesity, deprescribing, salt

## Abstract

Hypertension is the second biggest known global risk factor for disease after poor diet; perhaps lifestyle interventions are underutilized? In a previous small pilot study, it was found that a low carbohydrate diet was associated with significant improvements in blood pressure, weight, ‘deprescribing’ of medications and lipid profiles. We were interested to investigate if these results would be replicated in a larger study based in ‘real world’ GP practice. 154 patients with type 2 diabetes or impaired glucose tolerance were recruited into an observational cohort study in primary care. The effects of a low carbohydrate diet sustained for an average of two years (interquartile range 10–32 months) on cardiovascular risk factors were examined. Results demonstrate significant and substantial reductions in blood pressure (mean reduction of systolic BP 10.9 mmHg (interquartile range 0–22 mmHg) (*p* < 0.0001), mean reduction in diastolic BP 6.3 mmHg (interquartile range 0–12.8 mmHg) (*p* < 0.0001) and mean weight reduction of 9.5 Kg (interquartile range 5–13 Kg) (*p* < 0.0001) together with marked improvement in lipid profiles. This occurred despite a 20% reduction in anti-hypertensive medications. This novel and potentially highly effective dietary modification, done very cheaply alongside routine care, offers hope that should be tested in a large prospective trial.

## 1. Introduction

The National Institute for Health and Care Excellence (NICE) defines hypertension as sustained clinic blood pressure of 140/90 mmHg or higher and either a subsequent ambulatory blood pressure monitoring daytime average or home blood pressure monitoring average of 135/85 mmHg or higher. According to a recent study on disease [1], high blood pressure is the second biggest known global risk factor for disease after poor diet. In the UK, high blood pressure is the third biggest risk factor for disease after tobacco smoking and poor diet.

Most individuals with hypertension have no readily identifiable cause and are termed “essential” hypertensives. Many health care professionals are unaware of what the causation may be beyond vague references to salt and obesity. Hypertension is likely caused by a combination of salt and volume retention [2], together with neuroendocrine dysfunction. It is perhaps surprising the mechanisms which initiate and sustain high blood pressure remain unclear despite a century of intensive research. The role of salt (sodium) retention is probably central but uncertain regarding the exact mechanisms. 

In 2013 the authors started offering advice on a low carbohydrate diet (defined as <130 g carbs/day) to help people in the Norwood primary care (GP) practice with type 2 diabetes (T2D) and pre-diabetes or impaired glucose tolerance (IGT) [3]. In this eight-month pilot study of 19 patients the authors were struck by the significant and unexpected improvements in blood pressure (systolic 148 ± 17 to 133 ± 15 mmHg, *p* < 0.005; and diastolic 91 ± 8 to 83 ± 11 mmHg, *p* < 0.05). This occurred despite discontinuing several antihypertensive drugs. 

Our hypothesis for the 2013 pilot study was that a low carbohydrate diet may offer better diabetes control and weight management, compared to baseline results achieved with ‘standard care’. It is surprising now (in 2019) that in 2013 this was a controversial suggestion. Since 2013 this approach has gained wider acceptance [4,5].

Improvements in features of the metabolic syndrome associated with a reduction in carbohydrate intake had been demonstrated as early as 2005 and considered to be mediated by an effect on insulin activity [6]. A reduction in not just sugar but all dietary sources of glucose such as bread, potatoes and cereals reduces both insulin secretion and improves insulin sensitivity. Insulin resistance is implicated in the origins of the metabolic syndrome, which includes the following: Blood pressure, T2D, central obesity, dyslipidaemia and non-alcoholic fatty liver disease (NAFLD) (6). In 2013, a relevant meta-analysis of randomised controlled trials (>12 months duration) [7] comparing very-low-carbohydrate, ketogenic diets with low-fat diets for long-term weight loss found significant improvements in diastolic pressure but not systolic blood pressure. In the same year, a randomised controlled trial [8] found that compared to baseline, both systolic and diastolic blood pressure decreased after six weeks (*p* < 0.01).

Hyperinsulinaemia in people with T2D promotes renal sodium retention, a process not seen in non-diabetic people [9,10,11]. In 2017, a systematic review and meta-analysis of randomized control trials [12] concluded that a lower glycemic diet may lead to important reductions in blood pressure. It is possible that this improvement could be mediated through the loss of sodium mediated by a lower carb diet, rather than weight loss alone. This may offer intriguing insights into the role of dietary salt and its effect on blood pressure.

Despite the wider acceptance mentioned above, questions remain about the low carb approach. For example the British Diabetic Association in their November 2018 policy statement [13] whilst accepting a low carb diet can be effective ‘short term’ in managing weight, improving glycaemic control and cardiovascular risk in people with Type 2 diabetes feel that more research is needed to determine the effect of long-term adherence (over 12 months) on ‘heart health’.

The significant improvements in blood pressure in our initial pilot made us wonder firstly, if a larger cohort advised on a low carbohydrate diet for longer than six months would confirm our findings, and secondly what the possible mechanism for blood pressure (BP) improvement could be. Is it simply weight loss or something more?

Outcomes to be studied:

Primary, to examine the impact of dietary carbohydrate restriction on blood pressure and weight.

Secondary, to examine the impact of dietary carbohydrate restriction on lipid profiles and antihypertensive drug prescribing.

## 2. Materials and Methods

We performed a retrospective analysis of clinical data from routine work from the Norwood Surgery, a suburban GP practice with 9700 patients in the North of England.

**Figure 1:**
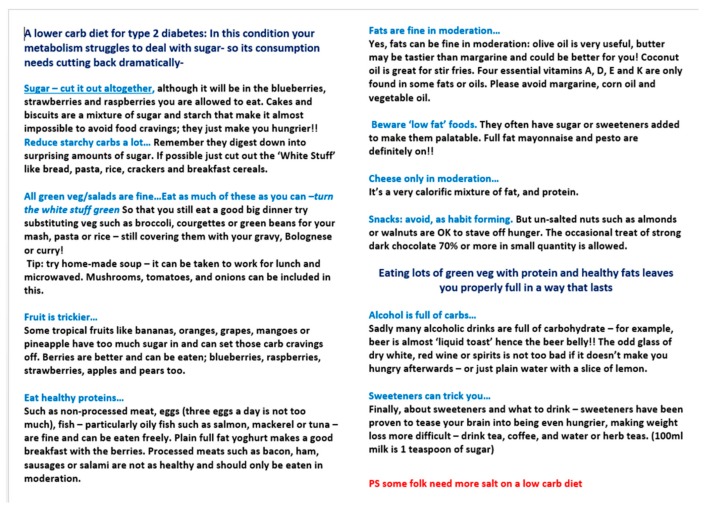
The standard Norwood Surgery low carb diet sheet.

A low carb diet was offered as part of routine care by GPs and practice nurses to practice patients with type 2 diabetes (T2D) or impaired glucose tolerance (IGT). Exclusion criteria were end of life patients, pregnancy, eating disorders, being underweight, type 1 diabetes or being under 18 years of age. People with type 2 diabetes on insulin were included (*n* = 6). Informed consent was obtained. Data was collected from March 2013 to November 2018 during which time we offered patients our study diet programme as part of ordinary appointments. Those who chose to enroll were given our diet sheet (Figure 1) and a review appointment a few weeks later to answer practical questions. Further support was offered depended upon patient choice and clinical need. This covered quite a broad spectrum from generally well people with IGT to eighty-year old persons on insulin, reflecting the fact this was part of our day to day work. However, in addition to ‘one to one’ doctor or nurse appointments we offered regular 90 min ‘group sessions’ for up to 30 people at approximately monthly intervals. This included family members, particularly if they did the shopping or cooking. Our cohort consisted of 154 patients, 90 men and 64 women for whom we had complete data sets for our primary outcomes of blood pressure and weight. Of the 154 participants, 89 were coded as having T2D. This represented 19% of the total practice population with T2D. The age range was 40–89 with a mean of 63 years at the onset of participation (inter-quartile range 53–73 years). The participants tended to be overweight starting with a mean BMI of 34 kg/m^2^ and a weight of 95.2 Kg (IQR 82.5–105.5 Kg).

Baseline measurements included the following: Weight, blood pressure, total cholesterol, HDL cholesterol, fasting triglyceride levels and medications for hypertension. All measurements were collected using standard UK National Health Service equipment and laboratory analysis.

**Figure 2:**
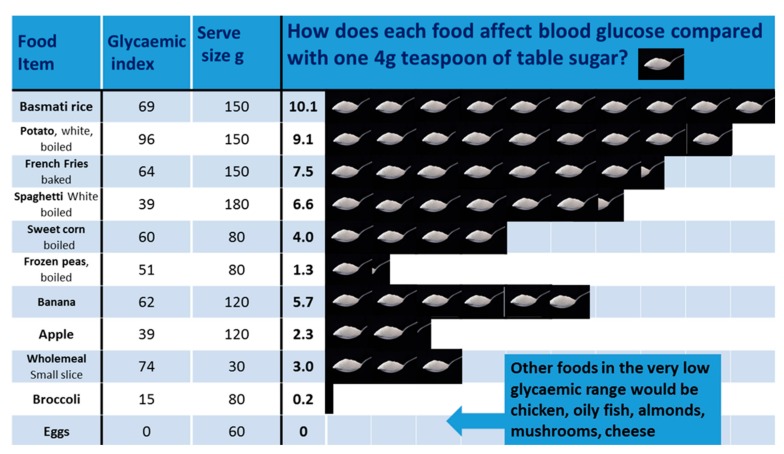
Patient friendly infographic to represent the glycaemic load of various foods.

We concentrated particularly on advising a dramatic reduction in total dietary sugar, explaining that this involves not just cutting back on table sugar itself but starchy carbohydrates like bread, cereals and potatoes that are themselves made up of glucose (in the form of starch). To help, we used our novel model based on a teaspoons of sugar equivalance system which represents the glycaemic load of various foods [14]. For example, one small slice of brown bread causes the same rise in blood glucose as three teaspoons of table sugar. Another example would be that a 150 g bowl of boiled rice will affect blood sugar to the same extent as ten teaspoons of table sugar. Information such as this helped patients understand that a breakfast of cornflakes, brown toast and apple juice is, in effect, sugar then more sugar. Our novel teaspoon of sugar equivalence system was used to produce a set of seven patient-friendly infographics (see for example Figure 2). These represent the glycaemic load of portions of food in terms of teaspoons of sugar and have been endorsed by NICE (March 2019) as suitable resources for helping adults with Type 2 diabetes make better dietary choices.

Patients also found it helpful to understand the physiological link between glucose, insulin and obesity, where insulin could be seen as ‘pushing’ excess glucose into fat cells. This advice was reiterated with our basic diet sheet (Figure 1) and a brief explanation of the low carb diet as a possible route to weight loss and better diabetes control or even medication-free remission. The sheet advocated matching substantial cuts in carbohydrates with an increased intake of green vegetables, a moderate increase in whole (non-juiced), low Glycaemic index fruits and ‘healthy fats’ found in olive oil, butter, eggs, nuts, full-fat plain yoghurt and some dairy products. Patients were also encouraged to eat non-processed meat and fish. Some found it helpful to think of replacing the ‘white stuff’ like potatoes, bread or rice with ‘green stuff’ like broccoli, spring cabbage or courgette. Weighing of food or calorie counting was not advised as we found this less sustainable.

A rough assessment of follow up would give an average review frequency of 6 per year, including the group sessions. One novel way to monitor compliance and help participants refine their diet was to ask them to use their mobile phone to photograph everything they ate and drank for the two days before their review. We used this particularly for those individuals who were disappointed with their weight loss. Others preferred to recount this information verbally. Because some of the patients were being followed up for to six years it was important to review the basic diet sheet with participants at least once a year rather than assume it had all been remembered. In addition to dietary measures, personal goal setting and the use of relevant patient feedback such as weight or blood tests results were emphasized as all participants were informed of their test results.

**Statistical methods.** Non-normally distributed continuous variables (blood pressure, lipid values, Hba1c and weight) are shown as mean values with the associated inter-quartile range (25th percentile, 75th percentile). Categorical variables (number of patients) are shown as absolute values with the percentage (%). Comparisons between continuous variables are made using the Wilcoxon Signed Rank test for paired samples. Baseline and follow-up distributions of patient data are presented visually using box and whisker charts, the upper and lower box lines on these charts indicate the inter-quartile range, the line in the middle of the box indicates the median value, the dot indicates the mean value and the upper and lower whiskers indicate either 1.5 times the interquartile range or the minimum/maximum value, whichever is the smaller distance from the box. Statistical analyses were performed with R version 3.4.0. In all cases a *p*-value < 0.05 was considered statistically significant.

## 3. Results

**Figure 3:**
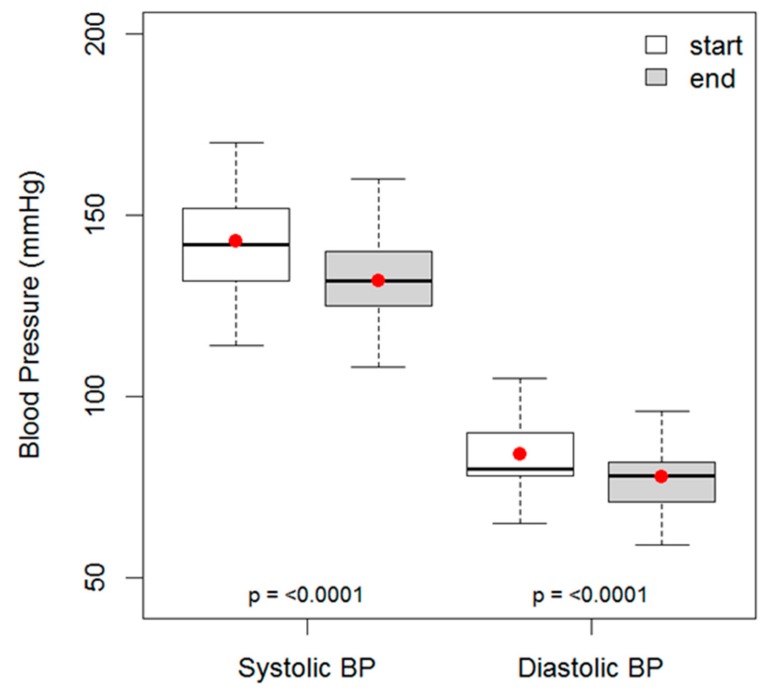
Box and whisker plots to show the distributions of the 154 patients’ systolic and diastolic blood pressure results before and after an average of 24 months on a low carb diet.

**Figure 4:**
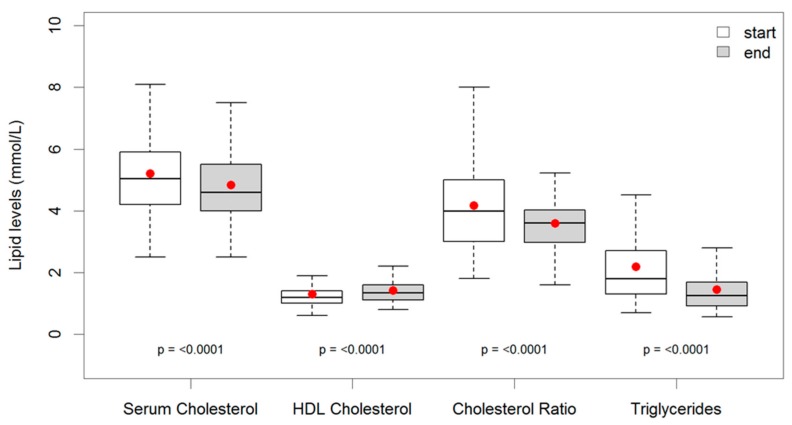
Box and whisker plots of the lipid profile results before and after an average of 24 months on a low carb diet. (Serum cholesterol is total cholesterol, triglyceride is a fasting sample).

The mean time spent on the diet was 24 months (IQR 10–32 months). This was associated with a mean unadjusted reduction of systolic BP of 10.9 mmHg (IQR 0–22 mmHg) (*p* < 0.0001) and a mean fall in diastolic BP of 6.3 mmHg (IQR 0–12.8 mmHg) (*p* < 0.0001), see Figure 3. The total cholesterol, HDL cholesterol, and fasting serum triglyceride levels are shown in Table 1 and Figure 4. Total cholesterol fell by 0.4 mmol/L (IQR −0.1–0.7), and serum triglyceride by 0.7 mmol/L (IQR 0.1–1.1). An overall sustained reduction of 8% in mean cholesterol and a very large reduction of 32% in mean TG. HDL rose 8% over the duration of the study. The mean total cholesterol:HDL ratio improved from 4.0 to 3.4, a 15% improvement.

The blood lipids all show significant results but the improvements in serum triglyceride levels at 32% were particularly striking. 

**Figure 5:**
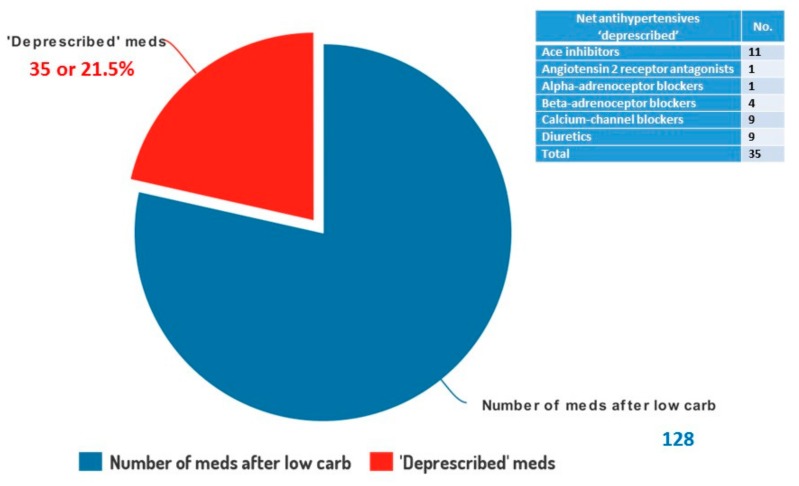
Pie chart to show the number of medications for hypertension deprescribed for the 154 patients on a low carbohydrate diet over an average of 24 months.

The 154 patients began the study on a total of 163 drugs for hypertension, by the end that had dropped to 128 as there had been a net ‘deprescribing’ of 35 repeat medications, which represented 21.5% of the total (see Figure 5). The two most commonly deprescribed drugs were Perindopril (11) and Amlodipine (9).

Mean weight fell overall by 9.5 kg (IQR 5–13) (*p* < 0.0001) a 10% reduction.

Data collection for lipids was less complete, reflecting the prospective ‘real life’ environment of this study where patients did not all agree to having blood tests, and of those who did some found the fasting sample necessary for a triglyceride level inconvenient. 

## 4. Discussion

The Norwood diet, based on a recommendation for a substitution of carbohydrates with lower carb alternatives, enabled substantial and sustained improvements in the measured variables of weight, blood pressure and lipid parameters. With an average participation of two years our study, it was of long duration compared to many published dietary studies [7,8]. Though in 2008, Nielsen [15] showed improvements in lipid profile which were still significant after 44 months on a low carbohydrate diet (blood pressure was not mentioned). 

The effects on blood pressure are particularly interesting. It is likely the improvements underestimate the actual reduction in hypertension burden as 27 patients were able to reduce or discontinue antihypertensive therapy. Their BP at the start could be said to be ‘falsely’ low as they were on medications discontinued by the end of the study. To correct for this we could re-calculate the averages by adjusting the baseline BP to what it was before the medication was started in those 27 individuals. On doing this we arrive at an adjusted BP lowering effect for the cohort of 14.8 mmHg systolic and 8.1 mmHg diastolic. 

A reduction of body weight by 9.5 kg would be predicted to lower BP by about 10 mm Hg systolic. This “one mmHg for one kg” has been recognised for 20 years and is a predictable effect [15]. It is interesting to speculate on the actual physiological mechanism linking obesity to high blood pressure. Harsha [16] suggests that reductions in insulin resistance, enhanced sodium retention, alterations in vascular structure and function, changes in ion transport, enhanced stimulation of the renin-aldosterone-angiotensin system, increased activation of sympathetic nervous system, and changes in natriuretic peptide all may play a part. However, he raises an important point; the possibility that the effects of weight loss are mediated through some other system and that weight loss itself might not be an independent influence on blood pressure status. He goes on to speculate that it is possible that some aspects of diet, when altered, are the true determinants of blood pressure reduction.

Weight loss alone cannot explain our adjusted drop in blood pressure. Some of the hypertensive patients who responded to the low carbohydrate diet were not even overweight at the beginning. For example, one 50 year-old lady had poorly controlled hypertension of 142/94 mmHg whilst taking perindopril 4 mg daily despite a ‘healthy’ BMI of just 22.1. Ordinarily it would never have occurred to us to advise any kind of diet for this patient but she was interested to try due to her abnormal liver function tests that suggested non-alcoholic fatty liver disease. Over a few months of a low carb diet her BP improved to 132/75 mmHg, even after stopping the perindopril (an improvement that has lasted three years so far). She also lost 2 kg in weight. It is possible the physiology of insulin’s action on the kidneys to cause sodium retention holds a clue in cases like these. As mentioned in the introduction, a low carb diet may improve both hyperinsulinaemia and insulin resistance, promoting the loss of sodium in the urine so improving blood pressure. This may explain our (adjusted) average 1.6 mm Hg reduction in systolic blood pressure per 1 kg reduction in weight. An effect which is significantly larger than predicted by the average observed weight loss alone.

In relation to the above theory around BP, insulin and sodium retention, by far the most convincing and tested theory is that seen in randomised controlled trials utilising the new SGLT2 inhibitor drugs which act on the renal sodium-glucose co-transport2 system [17]. These appear to improve multiple hard endpoint outcomes across diabetes, cardiovascular disease and renal medicine [17,18]. Looking at this in more depth, hyperglycemia causes hyperfiltration and the intra-glomerular pressure to rise [19,20]. Virtually all glucose filtered at the glomerulus is reabsorbed in the proximal tubule, up to a limit of around 180 mg/dL [19]. Above this level the maximal reabsorptive capacity is breached and excess glucose appears in the urine. SGLT2 is responsible for 80–90% of this reabsorption, with SGLT1 (a related transport system) in the distal tubule responsible for the remainder (10–20%) [19]. Hyperinsulinaemia augments the expression of SGLT2 in a dose-dependent manner and therefore the capacity within this system, which promotes a vicious cycle of increased ability to ‘save’ not just glucose but also sodium [20,21]. This, therefore, both maintains and exacerbates hyperglycaemia and sodium/fluid retention worsening diabetic control and hypertension. Moreover, hyperglycaemia disrupts delicate tubuloglomerular feeback mechanisms, reducing sodium delivery to the macula densa, mimicking kidney hypo-perfusion [19,20]. This leads to afferent arteriolar dilatation and efferent arteriolar constriction, worsening intra-glomerular hypertension and hyperfiltration [19,20]. SGLT2 inhibitor drugs (Emapagliflozin and Canagliflozin) reverse the above mechanisms, preventing sodium and glucose reabsorption and allowing normal tubuloglomerular feedback to occur [17,18,19,20]. It can therefore be hypothesised that reducing hyperinsulinaemia and hyperglycaemia by low carbohydrate dietary strategies, naturally reduces SGLT2 expression and up-regulation in the kidney, without the need for drugs. SGLT2 drugs are known to cause an osmotic diuresis and a contraction in plasma volume, estimated to be around 7% [16,17,18,19]. This diuretic effect was mentioned by our patients as it is also seen in low-carbohydrate diets with a certain amount of “water-weight” being lost in the initial stages [22]. 

The overall blood pressure reduction by 11/6 mmHg, if sustained over years would lower stroke risk by >30% and cardiovascular death by >20% [23]. Moreover, a reduction in cholesterol of 0.4 mmol/L, together with improvements in triglycerides and HDL, would be predicted to reduce CV events by approximately 10% if maintained long-term [24] 

Can a low carbohydrate diet be maintained long term? Many of the often-quoted studies about diet and health are surprisingly short term. Perhaps most notably, the widely cited DASH diet [25] to lower blood pressure, referenced in every hypertension guideline, was a six-week study in 412 people. In our own study, the subjects were happy to continue the dietary change for an average of two years (so far). Their motivation to continue seemed to be linked to a number of factors. Firstly, it seemed to help if they had clearly defined goals, these were often around weight loss and better control of diabetes. Secondly feedback also helped motivation, this was provided by the various parameters we were measuring. GP computer systems can generate graphs for weight or lipid results easily. These graphs proved very popular with our patients. One observation that participants made repeatedly was how surprised they were not to feel hungry. A relevant review on carbohydrates and appetite [26] concluded that, relative to low-GI carbohydrates, consumption of otherwise similar but high-GI carbohydrates promotes a more rapid return of hunger and increases subsequent energy intake. 

Limitations of the study. We present a simple cohort study taken from the ‘real world’ of 10 min GP appointments. As such it has low internal validity as there were many variables we were not able to control. For example, these 154 people were not randomly generated. At the very least they all chose to take part rather than being randomly allocated. However, we hope that its basis in an ordinary GP practice gives at least the possibility of greater external validity in its potential to be replicated in other practices. In any case, dietary RCTs, particularly longer-term studies of over a year, may well suffer from the problem that diet is a very personal thing. It is unlikely that many people would be prepared to eat a diet long-term that they have not chosen for themselves. Another problem we faced, common to dietary research generally, is assessing adherence to the diet. What foods did participants actually consume? In part this was addressed by regular ‘revision’ of the physiological principles around insulin and the diet sheet itself. Our research grant totaled just £7000 for a study lasting six years. Even with a large grant we could never know with certainty what 154 people eat over a period of years. This problem was highlighted by the Observing Protein and Energy Nutrition (OPEN) Study [27]. Doubly labeled water and urinary nitrogen was used to assess the accuracy of data from self-reported diet questionnaires, 35% percent of men and 23% of women were defined as under reporters of both energy and protein intake. Rather than attempt the impossible with probably flawed food diaries, we decided it was more practical to measure agreed outcomes having given standard advice as per Figure 1. In this way, we attempted to answer the question ‘what outcomes may a clinician expect if giving advice about our approach to people with type 2 diabetes and impaired glucose tolerance?’ Despite this, patients told us repeatedly they were eating far less sugar and starchy carbs. The significant weight loss measured suggests it is likely they were telling the truth. Finally, in this paper we have not reported on any measures of diabetic control as this is the subject of a separate piece of work. 

One of the striking features of our clinical work has been revealed by asking patients how they feel about long-term medication for blood pressure. When offered the choice of starting medication or buying in to lifestyle change with practice-based support, not a single patient opted to start the medication in six years. Similarly, we found patients were excited by the possibility of discontinuing medication they previously had assumed was ‘for life’. 

Our results may help clinicians uncertain how to best help a patient with type 2 diabetes, high blood pressure and a significantly raised triglyceride with a low HDL cholesterol level. A common scenario as our study shows. In this situation many clinicians may choose lifelong treatment with an SGLT2 inhibitor drug. From our work, it would seem reasonable to offer patients the alternative of a low carb diet which appears to offer similar benefits as well as cost savings and fewer side effects. For the low carb approach, the 32% improvements in serum triglyceride were particularly impressive. Indeed, emerging research from other sources is now associating improvements in cardiovascular health with a low carbohydrate diet. A recent systematic review and meta-analysis [28] concluded that large randomized controlled trials of at least six months duration with carbohydrate restriction appear superior in improving lipid markers when compared with low-fat diets. Furthermore, a new meta-analysis [29] (though merely epidemiological) suggests a possible association between high carbohydrate intake and increased cardiovascular risk where the authors suggest a 44% increase in coronary heart disease risk for every additional 65 grams of glycaemic load per day.

We believe it is likely that the significant effect on blood pressure results was in part due to a reduction in insulin, together with improved insulin sensitivity. As discussed in the introduction, it is well known that high insulin levels elevate blood pressure, promote myocardial hypertrophy and increase sodium retention by the kidney. The marked and sustained improvements in our cohort could be explained by the effects of a low carbohydrate diet on insulin action in T2D and IGT patients, where insulin levels are high and insulin resistance marked. Sodium has been demonised as the cause of hypertension when perhaps insulin and insulin resistance may actually be the culprits. The improvements in blood pressure were despite 27 patients being able to reduce or discontinue antihypertensive therapy by a net total of 35 medications, a 21% reduction in prescribing of drugs for essential hypertension for the cohort. This raises the additional possibility of substantial drug budget savings from a lower carb approach to hypertension.

Next steps:

Our study, carried out in general practice in the UK, lacks data on changes in insulin sensitivity and resistance. We plan to address this in a further pilot study with accurate determination of insulin activity before and after a sustained period on the Norwood diet. We hypothesise that there will be a substantial improvement in insulin sensitivity and a reduction in post prandial insulin levels, and we look forward to this next investigation. Clearly, confirmation of our findings in a large prospective study is crucial.

The Global Burden of Disease, Injuries and Risk Factor Study [1] looked at attributable disease risk factors in 188 countries. They conclude; behavioural, environmental and occupational, and metabolic risks can explain half of global mortality. In 2013, they felt dietary risks accounted for 11.3 million deaths and high systolic blood pressure for 10.4 million deaths. The magnitude of the suffering this data represents makes further studies vital, but the problem remains as to how this research is to be funded, particularly as the solutions to these global epidemics are in our opinion, unlikely to be pharmaceutical.

## 5. Conclusions

Adherence amongst people with T2D or glucose-intolerance to a low carbohydrate diet for an average of two years resulted in significant improvements in blood pressure, weight and lipid parameters despite ‘deprescribing’ of 21.5% of the total drugs for hypertension. The diet was well tolerated. These substantial benefits may translate into significant cardiovascular protection and drug budget savings that should be tested in a large prospective trial. Restricting dietary carbohydrates is now recognised as one way to help control the international epidemic of T2D [5]. Our work helps highlight how this simple dietary approach may also bring additional and so far under reported improvements to the increased cardiovascular risks that people with type 2 diabetes and impaired glucose tolerance face.

## Figures and Tables

**Table 1:** Biometrics before and after an average of 24 months on a low carbohydrate diet.

Biometric	Baseline Mean	Latest Follow up Mean	Improvement Mean (IQR)	p Value	Matched Pairs n (%)
**Weight** in Kg	**95.2**	**85.7**	**9.5** (5.0, 13.0)	**<0.0001**	**154** (99)
**Serum cholesterol**	**5.2**	**4.8**	**0.4** (−0.1, 0.7)	**<0.0001**	**110** (71)
**HDL cholesterol**	**1.3**	**1.4**	**0.1** (−0.1, 0.2)	**<0.0001**	**116** (75)
**Triglyceride**	**2.2**	**1.5**	**0.7** (0.1, 1.1)	**<0.0001**	**87** (56)
**Systolic BP** mmHg	**143**	**132**	**10.9** (0.0, 22.0)	**<0.0001**	**154** (99)
**Diastolic BP** mmHg	**84**	**78**	**6.3** (0.0, 12.8)	**<0.0001**	**154** (99)

The average time on the diet was 24 months (interquartile range 10–32 months).
